# Central injection of epidermal growth factor (EGF) induces hypophagia via D₁ dopaminergic and β₂ adrenergic receptors in broiler chickens

**DOI:** 10.1016/j.psj.2025.106058

**Published:** 2025-11-02

**Authors:** Zahra Jafari-Ardakan, Morteza Zendehdel, Kimia Mahdavi

**Affiliations:** Department of Basic Sciences, Faculty of Veterinary Medicine, University of Tehran, Tehran, Iran

**Keywords:** Appetite regulation, Epidermal Growth Factor, Dopamine receptor, Adrenergic receptor, Neonatal chick

## Abstract

In avian species, the central nervous system orchestrates feeding behavior through intricate interplay among diverse neurotransmitter pathways. Epidermal Growth Factor (EGF), a key peptide in cell growth and repair, has been implicated in appetite control in mammals, but its role in avian species remains unexplored. The present study was conducted to investigate the effect of central infusion of EGF on food consumption in neonatal broilers and to determine the possible involvement of dopaminergic and adrenergic systems. In a series of experiments, 5-day-old male broilers were subjected to intracerebroventricular (ICV) injections following a 3-hour fasting period. In experiment 1, chicks received an ICV injection of either the control solution or EGF at doses of 50, 100, or 200 ng. Experiments 2-10 evaluated the effects of co-injection of EGF (200 ng) with various pharmacological agents and receptor antagonists: SCH23390 (D₁-dopaminergic), AMI-193 (D₂-dopaminergic), l-DOPA (dopamine precursor), 6-OHDA (dopaminergic neurotoxin), prazosin (α₁-adrenergic), yohimbine (α₂-adrenergic), metoprolol (β₁-adrenergic), ICI 118,551 (β₂-adrenergic), and SR 59230R (β₃-adrenergic). At 30, 60, and 120 minutes after the infusion, cumulative food consumption was recorded. The results showed that EGF (100 and 200 ng) significantly and dose-dependently reduced cumulative feed consumption compared to the control group (*P* < 0.05). The anorexigenic effect of EGF (200 ng) was potentiated by co-injection with l-DOPA (*P* < 0.05). Conversely, the anorexigenic effect of EGF was attenuated by co-infusion of SCH23390, 6-OHDA, and ICI 118,551 with EGF (*P* < 0.05). Antagonists for α₁, α₂, β₁, and β₃ adrenergic receptors, as well as D₂ dopaminergic receptors, had no significant effect on EGF-induced anorexia (*P* ≥ 0.05). These findings demonstrate, for the first time, that central EGF acts as an appetite-suppressing peptide in broilers, and its effect is specifically mediated through interactions with D₁ dopaminergic and β₂ adrenergic receptor systems.

## Introduction

The control of feeding behavior constitutes a highly intricate biological mechanism orchestrated by the central nervous system (CNS), which integrates peripheral signals to maintain energy homeostasis (Kim et al., 2018). Despite shared characteristics in feeding behaviour between birds and mammals, significant differences exist in their underlying neurochemical pathways, underscoring the need for species-specific investigations (Roura and Foster, 2018). A detailed understanding of these mechanisms is particularly valuable for the poultry industry.

Beyond classical neurotransmitters, a growing body of evidence implicates growth factors in the central control of appetite (Dumbell, 2022). The 53-amino-acid polypeptide Epidermal Growth Factor (EGF), distinguished by its significant contributions to processes such as cell proliferation and tissue repair (Dao et al., 2018), has emerged as a potential modulator. Critically, a foundational study by Plata-Salamán (1988) demonstrated that intracerebroventricular (ICV) injection of EGF significantly suppressed short-term and nighttime food intake in rats, suggesting a direct central action of EGF on feeding regulation. EGF exerts its effects by binding to its specific tyrosine kinase receptor (EGFR) (Takeuchi and Ito, 2010). Importantly, the chicken EGFR (CER) has been cloned and characterized, showing high homology to the human EGFR, particularly in the tyrosine kinase domain (97 % identity), but with significant divergence in the extracellular ligand-binding region (Lax et al., 1988). A key finding from this work is that the avian receptor binds mammalian Transforming Growth Factor-alpha (TGF-α) with high affinity, while its affinity for mammalian EGF is approximately 100-fold lower. However, the expression of EGFR in avian hypothalamic appetite centers and the physiological role of central EGF signaling in avian feeding behaviour remain completely unknown, creating a significant gap in our understanding of comparative appetite neurobiology.

In parallel, the dopaminergic and adrenergic systems are well-established central regulators of food intake in chickens. For instance, central administration of dopamine and its precursor, l-DOPA, induces hypophagia primarily through D₁ receptors in broilers (Zendehdel et al., 2014). Similarly, the adrenergic system exerts complex, receptor-specific effects; norepinephrine injection in the paraventricular nucleus (PVN) suppresses feeding, while α₂-adrenergic receptor activation can stimulate it (Mahdavi et al., 2024). Metabolic studies suggest that EGF and adrenergic signals interact at multiple levels, including the modulation of cyclic AMP (cAMP) pathways. Specifically, EGF can inhibit the cAMP component of adrenergic signaling through activation of phosphodiesterases, thereby modulating metabolic responses to catecholamines (Grau et al., 1997). Recent molecular studies have demonstrated that β-adrenergic receptor activation can transactivate EGFR signaling pathways, promoting downstream kinases such as ERK1/2 and Akt associated with cell survival pathways (Grisanti et al., 2014). Moreover, dopamine has been shown to induce EGF release from neural precursor cells via metalloprotease-mediated cleavage of pro-EGF, thus linking dopaminergic signaling to modulation of EGF availability and EGFR activation (O’Keeffe and Barker, 2011).

Given the proven anorexigenic effect of central EGF in a mammalian model, the confirmed presence of a functional EGFR in chickens, and the compelling evidence for EGF-monoamine interactions, we hypothesized that centrally administered EGF would influence feed intake in broiler chickens. Furthermore, we postulated that this effect would be mediated through specific adrenergic and dopaminergic receptors, given their established role in avian appetite control. Thus, the study aimed to: 1) investigate the effect of ICV-injected EGF on meal consumption in neonatal broiler chicks, and 2) elucidate the potential involvement of specific α- and β-adrenergic, as well as D₁, and D_2_ dopaminergic receptor subtypes in mediating EGF's effects.

## Materials and methods

### Animals and housing

A total of 440 one-day-old broiler chicks (Ross 308) were procured from a commercial hatchery (Morghak Co., Iran). Upon arrival, the chicks were housed as a group under standard environmental conditions for an initial 2-day acclimatization period. The housing regimen consisted of a 23:1 hour light/dark cycle, a temperature maintained at 30 ± 1°C, and a relative humidity of 50 ± 5 %. Light intensity was maintained at 25–30 lux. After acclimation, birds were housed individually in wire cages (width: 30 cm, length: 40 cm, height: 40 cm), each equipped with a 10 cm linear feeder and a 250 mL water cup. On the third day post-hatch, the chicks were randomly distributed into individual experimental cages. All birds were provided with ad libitum access to fresh water and a standard starter diet, the detailed composition of which is presented in Table 1. Throughout the study, except during specified food deprivation periods, this dietary regimen was maintained. Prior to initiation, the study protocols received ethical review and approval from the University of Tehran Animal Ethics Committee (Approval Code: VET ETHIC-202509-1024). The investigation was carried out in strict conformity with the principles detailed in the NIH Guide for the Care and Use of Laboratory Animals, alongside all relevant national animal welfare legislation.

**Table 1:** Assessment of the components and nutrient profile of the diet used in the experiment.

Ingredient (%)		Nutrient analysis	
Corn	52.85	ME, kcal/g	2850
Soybean meal, 48 % CP	31.57	Crude protein (%)	21
Wheat	5	Linoleic acid (%)	1.69
Gluten meal, 61 % CP	2.50	Crude fiber (%)	3.55
Wheat bran	2.47	Calcium (%)	1
Di-calcium phosphate	1.92	Available phosphorus (%)	0. 5
Oyster shell	1.23	Sodium (%)	0.15
Soybean oil	1.00	Potassium (%)	0.96
Mineral premix	0.25	Chlorine (%)	0.17
Vitamin premix	0.25	Choline (%)	1.30
Sodium bicarbonate	0.21	Arginine (%)	1.14
Sodium chloride	0.20	Isoleucine (%)	0.73
Acidifier	0.15	Lysine (%)	1.21
DL-Methionine	0.10	Methionine (%)	0.49
Toxin binder	0.10	Methionine + cystine (%)	0.83
L-Lysine HCl	0.05	Threonine (%)	0.70
Vitamin D_3_	0.1	Tryptophan (%)	0.20
Multi enzyme	0.05	Valine (%)	0.78

Metabolisable energy (ME) and crude protein (CP) are expressed per kilogram of diet. The mineral supplement comprises 35.2 g of manganese sourced from MnSO₄∙H₂O; 22 g of iron derived from FeSO₄∙H₂O; 35.2 g of zinc obtained from ZnO; 4.4 g of copper from CuSO₄∙5H₂O; 0.68 g of iodine provided as ethylenediamine dihydroiodide; and 0.12 g of selenium from Na₂SeO₃. The vitamin supplement includes 1.188 g of retinyl acetate, 0.033 g of dl-α-tocopheryl acetate, 8.84 g of tocopherol, 1.32 g of menadione, 0.88 g of thiamine, 2.64 g of riboflavin, 13.2 g of nicotinic acid, 4.4 g of pantothenic acid, 1.76 g of pyridoxine, 0.022 g of biotin, 0.36 g of folic acid, and 1500 mg of choline chloride.

### Drugs

All chemical agents, including Epidermal Growth Factor (EGF) along with various receptor antagonists—SCH23390 (D₁), AMI-193 (D₂), prazosin (α₁), yohimbine (α₂), metoprolol (β₁), ICI 118,551 (β₂), and SR 59230R (β₃)—as well as l-DOPA (a dopamine precursor) and the neurotoxin 6-hydroxydopamine (6-OHDA), were acquired from Sigma-Aldrich (USA). Initially, each compound was solubilized in pure dimethyl sulfoxide (DMSO) before being diluted in a 0.85 % saline solution that contained 0.1 % Evans blue dye, yielding a final DMSO concentration of 1:250, a concentration previously established as non-neurotoxic (Blevins et al., 2002). The control solution was the mixture of saline/DMSO/Evans blue.

### Experimental design

Following a 3-hour fasting period (FD3), chicks were randomly allocated to various treatment groups (*n* = 11 per group). The research comprised ten distinct experiments, each incorporating four treatment groups (Table 2). The dosage selection for all pharmacological agents was based on established findings from prior studies in neonatal broiler chicks using the same ICV injection paradigm and fasting model, which confirmed these doses as effective or sub-effective on feed intake (Zendehdel et al., 2014, 2017; Baghbanzadeh and Hajinezhad, 2010).

**Table 2:** Injection protocols for experiments 1-10.

Experiments	Groups			
	A	B	C	D
1	Control solution	EGF (50 ng)	EGF (100 ng)	EGF (200 ng)
2	Control solution	SCH 23390 (5 nmol)	EGF (200 ng)	SCH 23390 + EGF (5 nmol) + (200 ng)
3	Control solution	AMI-193 (5 nmol)	EGF (200 ng)	AMI-193 + EGF (5 nmol) + (200 ng)
4	Control solution	L-DOPA (125 nmol)	EGF (200 ng)	L-DOPA + EGF (125 nmol) + (200 ng)
5	Control solution	6-OHDA (2.5 nmol)	EGF (200 ng)	6-OHDA + EGF (2.5 nmol) + (200 ng)
6	Control solution	Prazosin (10 nmol)	EGF (200 ng)	Prazosin + EGF (10 nmol) + (200 ng)
7	Control solution	Yohimbine (13 nmol)	EGF (200 ng)	Yohimbine + EGF (13 nmol) + (200 ng)
8	Control solution	Metoprolol (24 nmol)	EGF (200 ng)	Metoprolol + EGF (24 nmol) + (200 ng)
9	Control solution	ICI 118,551 (5 nmol)	EGF (200 ng)	ICI 118,551+ EGF (5 nmol) + (200 ng)
10	Control solution	SR 59230R (20 nmol)	EGF (200 ng)	SR 59230R + EGF (20 nmol) + (200 ng)

Control solution: saline/DMSO/Evans blue mixture; EGF: Epidermal Growth Factor; SCH 23390 (antagonist of D1-receptor); AMI-193 (antagonist of D2-receptor); l-DOPA (Dopamine precursor); 6-OHDA (dopaminergic neurotoxin), prazosin (antagonist of α₁-receptor), yohimbine (antagonist of α₂-receptor), metoprolol (antagonist of β₁-receptor), ICI 118,551 (antagonist of β₂-receptor), and SR 59230R (antagonist of β₃-receptor).

### Experiment 1: Dose-response assessment of EGF

This experiment aimed to determine the effective dose of Epidermal Growth Factor (EGF). Groups received ICV injections of either the control solution (CS), EGF (50 ng), EGF (100 ng), or EGF (200 ng).

### Experiments 2-10: Investigating the role of dopaminergic & adrenergic pathways

This series examined the contribution of dopaminergic and adrenergic signaling. The group structure for each experiment was as follows:

• Group 1: Control Solution (CS(
• Group 2: Sub-effective dose of the respective dopaminergic or adrenergic agent
• Group 3: Effective dose of EGF (200 ng)
• Group 4: Co-injection of EGF (200 ng) plus the respective dopaminergic or adrenergic agent

The specific agents and their doses for each experiment were:

• Experiment 2: SCH23390 (D₁ receptor antagonist, 5 nmol)
• Experiment 3: AMI-193 (D₂ receptor antagonist, 5 nmol)
• Experiment 4: l-DOPA (dopamine precursor, 125 nmol)
• Experiment 5: 6-OHDA (dopaminergic neurotoxin, 2.5 nmol)
• Experiment 6: Prazosin (α₁-adrenoceptor antagonist, 10 nmol)
• Experiment 7: Yohimbine (α₂-adrenoceptor antagonist, 13 nmol)
• Experiment 8: Metoprolol (β₁-adrenoceptor antagonist, 24 nmol)
• Experiment 9: ICI 118,551 (β₂-adrenoceptor antagonist, 5 nmol)
• Experiment 10: SR 59230R (β₃-adrenoceptor antagonist, 20 nmol)

This strategic approach, which combines effective and sub-effective doses of interacting agents, is designed to nullify opposing effects and thereby clarify potential functional interactions between the signaling systems under investigation.

### Injection procedure

ICV injections were performed according to a standardized stereotaxic technique (Furuse et al., 1997). Briefly, chicks were lightly restrained in an acrylic device without anesthesia, with the head fixed at a 45° angle. A microsyringe (Hamilton, Switzerland) was used to administer all solutions in a final volume of 10 µL. The injection site was targeted at the right lateral ventricle by drilling a guide hole through the skull and inserting the needle to a depth of 4 mm. This method is established to minimize procedural stress in neonates (Davis et al., 1979). To maintain temporal precision and minimize confounding variables, a strict staggered schedule was implemented for the injections. Birds were injected sequentially with a 3-minute interval between subjects. Immediately after the ICV injection, broilers were promptly placed back into their cages, where they had access to a known quantity of feed and clean water. Total feed consumption was evaluated at 30, 60, and 120-minute intervals after the procedure, with careful correction for any spilled food. To standardize the data, intake values were adjusted according to the body mass of each bird and are reported as grams of feed consumed per 100 grams of body weight (g/100 g BW).

Each bird was used only once. Upon completion of the measurements, chicks were euthanized via decapitation. The accuracy of the ventricular infusion was confirmed post-mortem by visualizing the the spread of Evans blue dye within the right lateral ventricle. The final dataset incorporated results solely from individuals for which successful injection placement was confirmed. The entire experimental timeline was restricted to the hours between 08:00 and 16:15.

### Statistical analysis

Findings are reported as the mean ± SEM. A two-way repeated measures ANOVA was employed to analyze cumulative feed intake, considering both treatment group and time as independent factors. In cases where statistically significant interactions were detected, multiple comparisons were carried out using Tukey's post hoc test. A probability value (P) of less than 0.05 was considered statistically significant. The statistical software SPSS (version 26) was used for all computations.

## Results

### Dose-response effects of EGF

**Fig 1:**
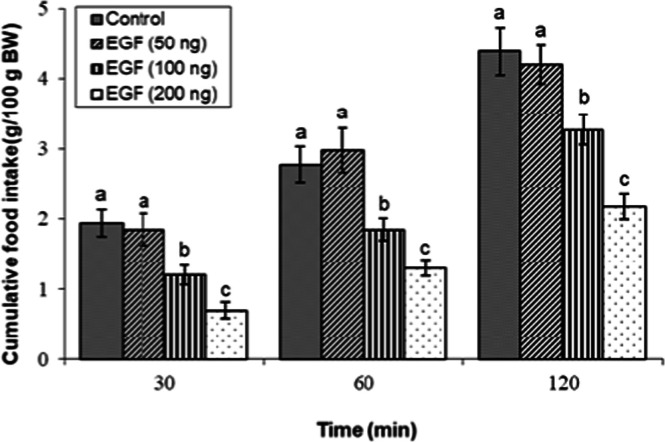
Evaluation of cumulative feed intake in broilers receiving ICV injections of vehicle or Epidermal Growth Factor (EGF) at doses of 50, 100, and 200 ng. Approved injections in each group: control (n = 11), EGF 50 ng (n = 8), EGF 100 ng (n = 11), and EGF 200 ng (n = 11). Values are means ± SEM. Statistical assessment confirmed significant main effects of treatment (F(3, 37) = 15.82, P < 0.001) and time (F(2, 74) = 185.43, P < 0.001), with a significant interaction effect (F(6, 74) = 3.01, P < 0.001). Means assigned different letters (a, b, c) differed significantly (P < 0.05) in Tukey's post-hoc analysis at individual time points.

ICV infusion of EGF induced a significant suppression of cumulative feed intake in FD3 chicks. Post-hoc analysis revealed that administration of EGF (100 and 200 ng) reduced feed consumption at all measured time points (*P* < 0.05). The 200 ng dose elicited the most potent hypophagic response. In contrast, the lowest dose of EGF (50 ng) did not produce a significant effect on feeding behavior (*P* ≥ 0.05; Fig. 1).

### Effects of dopaminergic & adrenergic agents alone

**Fig 2:**
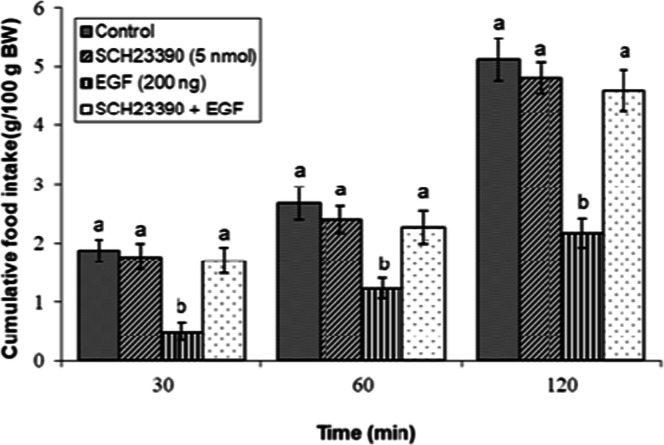
Evaluation of cumulative feed intake in broilers receiving ICV injections of vehicle, the D₁ receptor antagonist SCH23390 (5 nmol), EGF (200 ng), or both agents combined. Approved injections in each group: control (n = 10), SCH23390 (n = 11), EGF (n = 11), and combination (n = 11). Values are means ± SEM. Statistical assessment confirmed significant main effects of treatment (F(3, 39) = 22.15, P < 0.001) and time (F(2, 78) = 210.33, P < 0.001), with a significant interaction effect (F(6, 78) = 4.45, P < 0.001). Means assigned different letters (a, b) differed significantly (P < 0.05) in Tukey's post-hoc analysis at individual time points.

**Fig 3:**
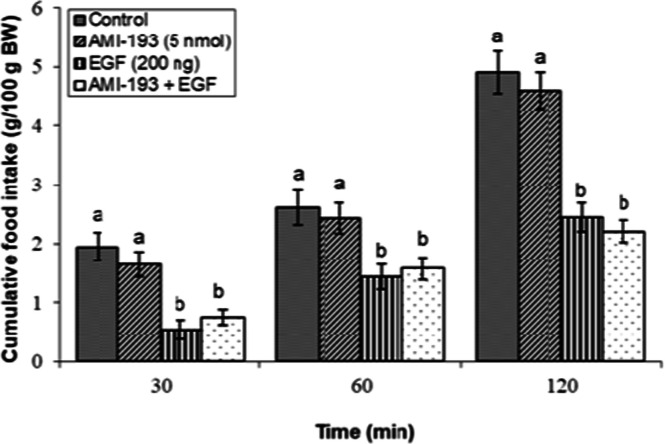
Evaluation of cumulative feed intake in broilers receiving ICV injections of vehicle, the D₂ receptor antagonist AMI-193 (5 nmol), EGF (200 ng), or both agents combined. Approved injections in each group: control (n = 11), AMI-193 (n = 9), EGF (n = 11), and combination (n = 10). Values are means ± SEM. Statistical assessment confirmed significant main effects of treatment (F(3, 37) = 18.92, P < 0.001) and time (F(2, 74) = 199.56, P < 0.001), with a significant interaction effect (F(6, 74) = 1.89, P < 0.001). Means assigned different letters (a, b) differed significantly (P < 0.05) in Tukey's post-hoc analysis at individual time points.

**Fig 4:**
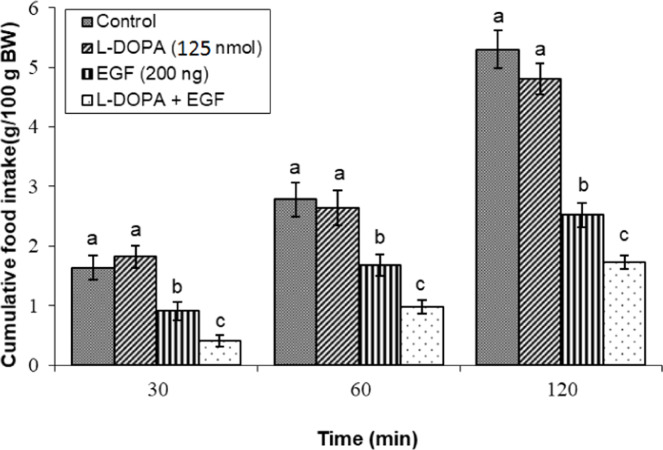
Evaluation of cumulative feed intake in broilers receiving ICV injections of vehicle, the dopamine precursor l-DOPA (125 nmol), EGF (200 ng), or both agents combined. Approved injections in each group: control (n = 10), l-DOPA (n = 11), EGF (n = 10), and combination (n = 11). Values are means ± SEM. Statistical assessment confirmed significant main effects of treatment (F(3, 38) = 25.67, P < 0.001) and time (F(2, 76) = 225.41, P < 0.001), with a significant interaction effect (F(6, 76) = 5.12, P < 0.001). Means assigned different letters (a, b, c) differed significantly (P < 0.05) in Tukey's post-hoc analysis at individual time points.

**Fig 5:**
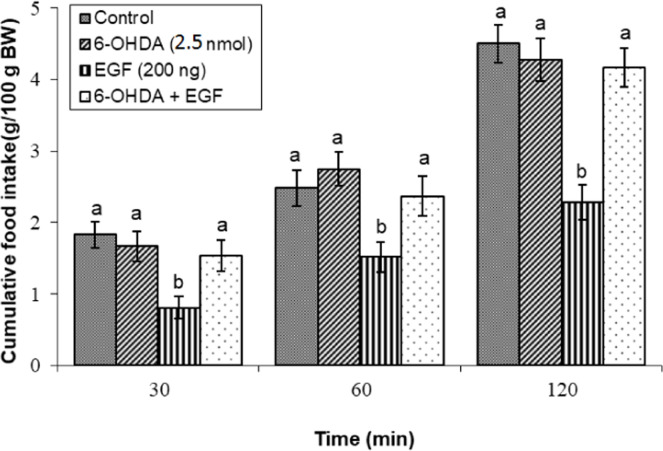
Evaluation of cumulative feed intake in broilers receiving ICV injections of vehicle, the dopaminergic neurotoxin 6-OHDA (2.5 nmol), EGF (200 ng), or both agents combined. Approved injections in each group: control (n = 11), 6-OHDA (n = 9), EGF (n = 11), and combination (n = 11). Values are means ± SEM. Statistical assessment confirmed significant main effects of treatment (F(3, 38) = 19.44, P < 0.001) and time (F(2, 76) = 188.92, P < 0.001), with a significant interaction effect (F(6, 76) = 3.45, P < 0.001). Means assigned different letters (a, b) differed significantly (P < 0.05) in Tukey's post-hoc analysis at individual time points.

**Fig 6:**
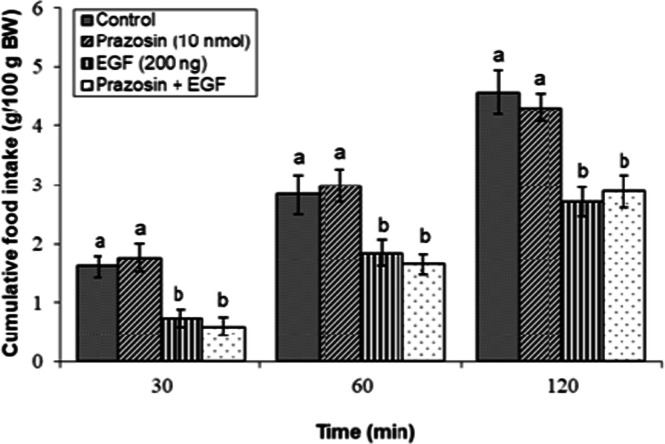
Evaluation of cumulative feed intake in broilers receiving ICV injections of vehicle, the α₁-adrenoceptor antagonist prazosin (10 nmol), EGF (200 ng), or both agents combined. Approved injections in each group: control (n = 8), prazosin (n = 11), EGF (n = 11), and combination (n = 10). Values are means ± SEM. Statistical assessment confirmed significant main effects of treatment (F(3, 36) = 16.78, P < 0.001) and time (F(2, 72) = 178.33, P < 0.001), with a significant interaction effect (F(6, 72) = 1.45, P < 0.001). Means assigned different letters (a, b) differed significantly (P < 0.05) in Tukey's post-hoc analysis at individual time points.

**Fig 7:**
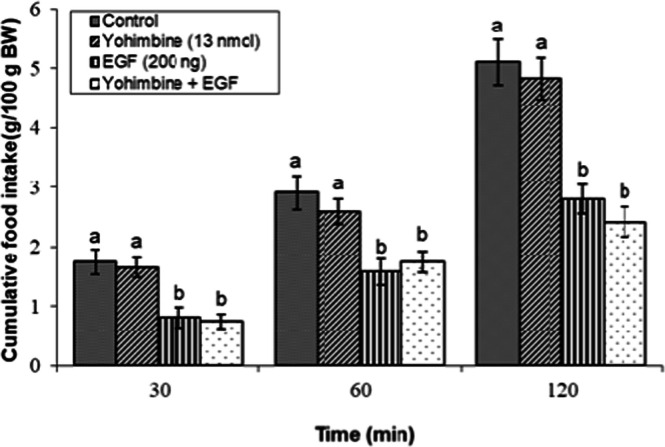
Evaluation of cumulative feed intake in broilers receiving ICV injections of vehicle, the α₂-adrenoceptor antagonist yohimbine (13 nmol), EGF (200 ng), or both agents combined. Approved injections in each group: control (n = 10), yohimbine (n = 10), EGF (n = 11), and combination (n = 9). Values are means ± SEM. Statistical assessment confirmed significant main effects of treatment (F(3, 36) = 17.21, P < 0.001) and time (F(2, 72) = 192.67, P < 0.001), with a significant interaction effect (F(6, 72) = 1.21, P < 0.001). Means assigned different letters (a, b) differed significantly (P < 0.05) in Tukey's post-hoc analysis at individual time points.

**Fig 8:**
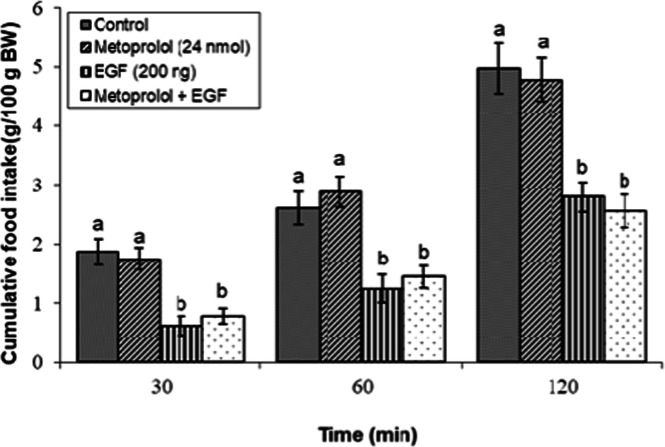
Evaluation of cumulative feed intake in broilers receiving ICV injections of vehicle, the β₁-adrenoceptor antagonist metoprolol (24 nmol), EGF (200 ng), or both agents combined. Approved injections in each group: control (n = 11), metoprolol (n = 11), EGF (n = 10), and combination (n = 8). Values are means ± SEM. Statistical assessment confirmed significant main effects of treatment (F(3, 36) = 15.99, P < 0.001) and time (F(2, 72) = 201.15, P < 0.001), with a significant interaction effect (F(6, 72) = 1.67, P < 0.001). Means assigned different letters (a, b) differed significantly (P < 0.05) in Tukey's post-hoc analysis at individual time points.

**Fig 9:**
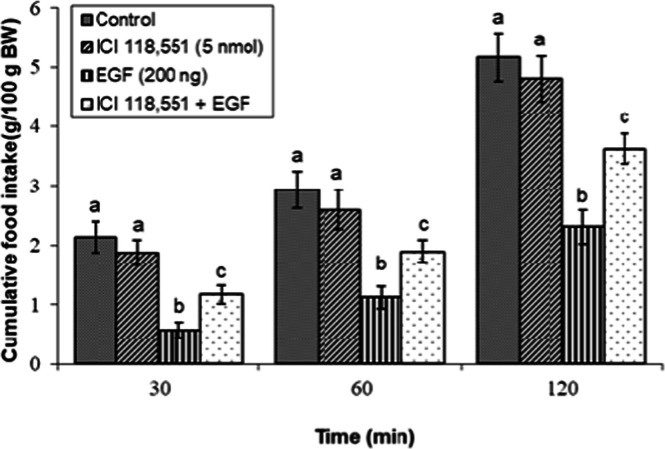
Evaluation of cumulative feed intake in broilers receiving ICV injections of vehicle, the β₂-adrenoceptor antagonist ICI 118,551 (5 nmol), EGF (200 ng), or both agents combined. Approved injections in each group: control (n = 11), ICI 118,551 (n = 11), EGF (n = 9), and combination (n = 9). Values are means ± SEM. Statistical assessment confirmed significant main effects of treatment (F(3, 36) = 20.11, P < 0.001) and time (F(2, 72) = 174.89, P < 0.001), with a significant interaction effect (F(6, 72) = 4.01, P < 0.001). Means assigned different letters (a, b, c) differed significantly (P < 0.05) in Tukey's post-hoc analysis at individual time points.

**Fig 10:**
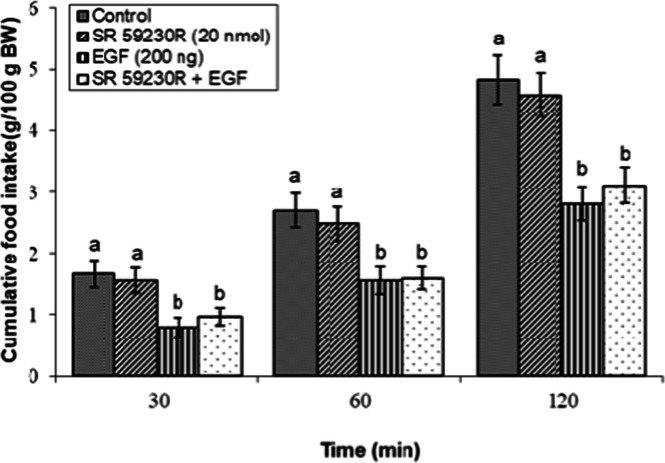
Evaluation of cumulative feed intake in broiler s receiving ICV injections of vehicle, the β₃-adrenoceptor antagonist SR 59230R (20 nmol), EGF (200 ng), or both agents combined. Approved injections in each group: control (n = 10), SR 59230R (n = 11), EGF (n = 10), and combination (n = 8). Values are means ± SEM. Statistical assessment confirmed significant main effects of treatment (F(3, 35) = 16.45, P < 0.001) and time (F(2, 70) = 165.34, P < 0.001), with a significant interaction effect (F(6, 70) = 1.88, P < 0.001). Means assigned different letters (a, b) differed significantly (P < 0.05) in Tukey's post-hoc analysis at individual time points.

ICV infusion of the selected sub-effective doses of dopaminergic receptor antagonists (SCH23390, AMI-193), the dopamine precursor l-DOPA, dopaminergic neurotoxin 6-OHDA, and adrenergic receptor antagonists (prazosin, yohimbine, metoprolol, ICI 118,551, SR 59230R) in the absence of EGF, did not alter cumulative feed intake consumption to the control solution (*P* ≥ 0.05; Fig 2, Fig 3, Fig 4, Fig 5, Fig 6, Fig 7, Fig 8, Fig 9, Fig 10).

### Interaction between EGF and dopaminergic pathways

The central dopaminergic system also contributed to EGF-mediated hypophagia. As illustrated in Fig. 2, the suppressive effect of EGF on feed consumption was markedly reduced by concurrent administration of the D₁-dopaminergic receptor antagonist SCH23390 (5 nmol) (*P* < 0.05). In contrast, ICV infusion of the D₂ receptor antagonist AMI-193 (5 nmol) failed to reverse EGF-mediated hypophagia, as the values obtained showed no significant deviation from those in the group that received EGF alone (*P* ≥ 0.05; Fig. 3).

Furthermore, potentiation of dopaminergic signaling via co-infusion of the dopamine precursor l-DOPA (125 nmol) with EGF resulted in a significant enhancement of the hypophagic response relative to EGF administration alone (*P* < 0.05; Fig. 4). Separately, the anorexigenic effect of EGF was also abolished by co-infusion with the dopaminergic neurotoxin 6-OHDA (2.5 nmol) (*P* < 0.05; Fig. 5).

### Interaction between EGF and adrenergic pathways

The hypophagia induced by the effective dose of EGF (200 ng) was significantly modulated by specific adrenergic receptor antagonists. As illustrated in Fig. 6, co-injection of the α₁-adrenoceptor antagonist prazosin (10 nmol) with EGF did not significantly alter feed consumption compared to EGF alone (*P* ≥ 0.05). Similarly, the anorexigenic response to EGF was not affected by concurrent administration of the α₂-adrenoceptor antagonist yohimbine (13 nmol; Fig. 7) or the β₁-adrenoceptor antagonist metoprolol (24 nmol) (*P* ≥ 0.05; Fig. 8).

In contrast, the suppressive effect of EGF on feeding was markedly attenuated by co-infusion of the β₂-adrenoceptor antagonist ICI 118,551 (5 nmol) (*P* < 0.05; Fig. 9). However, the β₃-adrenoceptor antagonist SR 59230R (20 nmol) failed to reverse EGF-induced hypophagia, as shown in Fig. 10 (*P* ≥ 0.05).

### Behavioral observations

No overt abnormal behaviors, such as sedation, ataxia, or barrel rotation, were observed following any of the ICV drug treatments, indicating that the observed effects on feed intake were not secondary to general motor impairment or distress.

## Discussion

This investigation reveals that centrally administered EGF dose-dependently inhibits feeding behavior in neonatal broilers. Furthermore, our pharmacological approach reveals that this hypophagic effect is specifically mediated via dopaminergic D₁ and adrenergic β₂ receptor pathways. EGF is a low molecular weight polypeptide composed of 53 amino acids (approximately 6400 Da), first identified for its ability to stimulate cellular proliferation and differentiation, particularly in epithelial tissues (Chen et al., 2016). The primary mechanism through which EGF elicits its biological actions involves binding to its specific receptor (EGFR), which is a transmembrane protein possessing intrinsic tyrosine kinase activity (Kaufman et al., 2021). Despite its well-characterized roles in cell growth and its implication in pathologies ranging from cancer to Alzheimer's disease (Boch et al., 2022; Jayaswamy et al., 2023), and although its anorexigenic effect following central administration has been documented in mammals (Plata-Salamán, 1988), its role in avian appetite regulation had remained unexplored prior to this investigation.

Our findings not only align with the seminal work of Plata-Salamán (1988), which established that ICV infusion of EGF suppresses food intake in rats, but they also unveil a novel and specific interaction between EGF signaling and monoaminergic systems in the avian brain. The conservation of EGF's hypophagic action across mammalian and avian species suggests a potentially ancient and evolutionarily conserved role for this growth factor in neural mechanisms governing energy balance.

A key consideration is the efficacy of mammalian EGF in chickens. Our results are consistent with the presence of a functional EGF receptor (CER) in the avian brain. While the CER exhibits a lower affinity for murine EGF than for ligands like TGF-α (Lax et al., 1988), the doses used in our study were sufficient to activate this pathway and modulate feeding, highlighting the importance of species-specific receptor pharmacology.

The most significant contribution of this study is the elucidation of the specific receptor mechanisms involved in EGF-induced hypophagia. The potentiation of EGF's effect by l-DOPA and its complete blockade by the D₁ antagonist SCH23390 strongly suggest a synergistic interaction or a downstream dependency on D₁ receptor activation. Additionally, the attenuation of EGF's effect by the dopaminergic neurotoxin 6-OHDA further supports the involvement of the dopaminergic system. This finding aligns with the well-established role of dopamine in translating motivational states into goal-directed feeding behavior across species (Barbano et al., 2009). In avian models, central administration of dopamine or l-DOPA consistently induces hypophagia (Zendehdel et al., 2014), and D₁ receptors have emerged as a pivotal nexus for integrating diverse anorexigenic signals. Previous studies in chicks have demonstrated that D₁ receptor blockade attenuates hypophagia induced by μ-opioid receptor agonists, serotonin, and cholecystokinin, and modulates hyperphagia induced by GABAA receptor activation (Ebrahimnejad et al., 2025; Hashemzadeh et al., 2018; Zendehdel et al., 2016). The specificity of this pathway is further highlighted by our results, as the hypophagia induced by EGF was attenuated by the dopaminergic neurotoxin 6-OHDA but not altered by antagonists for other dopamine receptor subtypes (D₂), underscoring the selective role of D₁ receptor-mediated signaling.

This observation gains further mechanistic insight from the work of Yoon and Baik (2013), who described a critical signaling cascade wherein dopamine D₂ receptor (D2R) stimulation leads to transactivation of the EGFR via ADAM10/17-mediated shedding of heparin-binding EGF (HB-EGF), subsequently activating the ERK pathway. Although our study implicates D₁ rather than D₂ receptors in an appetitive context, this established paradigm provides a compelling framework. It is plausible that central EGF administration modulates dopaminergic tone or D₁ receptor sensitivity through analogous intracellular cross-talk mechanisms, potentially involving EGFR-mediated enhancement of D₁ signaling efficacy within key hypothalamic circuits governing feeding. This potential cross-talk is further supported by the broader paradigm of ErbB-GPCR interaction, as highlighted by Sotoyama et al. (2023).

Similarly, the specific blockade of EGF's anorexigenic effect by the β₂-adrenergic antagonist ICI 118,551 unveils a novel and distinct role for this receptor subtype. The adrenergic system is a critical component of the central network regulating energy balance. Noradrenaline can modulate food intake through various receptor subtypes, with α₂-adrenoceptors often mediating orexigenic effects and β-adrenoceptors, particularly β₂, being linked to anorexigenic outcomes (Mahdavi et al., 2024). For instance, the α₂-adrenoceptor agonist clonidine stimulates feeding in chickens (Bungo et al., 1999), whereas its antagonist yohimbine can reverse noradrenaline's inhibitory effects on PVN cells, suggesting an orexigenic role for α₂ receptors (Wellman, 2000). Conversely, central administration of the β₂-adrenoceptor agonist salbutamol decreases food consumption in rats (Kanzler et al., 2011), a finding consistent with the general pattern in poultry where β₂ and β₃ receptor activation reduces intake (Baghbanzadeh and Hajinezhad, 2010). The complexity of this system is highlighted by the intricate interplay between adrenergic and other neurotransmitter pathways. Zendehdel and Hassanpour (2014) provided evidence that β₂ adrenergic receptors mediate ghrelin's anorexigenic action in chickens. Additionally, Zendehdel et al. (2017) found that the hypophagic response to serotonin involves signaling through both α₂ and β₂ adrenergic pathways.

Crucially, our results gain significant mechanistic and physiological context from documented functional interactions between the adrenergic system and EGF signaling in peripheral tissues. While our study focuses on central nervous system regulation, pioneering work has demonstrated a robust physiological axis where adrenergic stimulation triggers EGF release, which in turn modulates adrenergic responses. Specifically, Campreciós et al. (2009) demonstrated that acute α₁-adrenergic stimulation induces the secretion of EGF from submandibular salivary glands into the bloodstream in mice. This endogenously released EGF then acts on the liver to selectively attenuate the β-adrenergic component of adrenaline-induced glycogenolysis (Grau et al., 1997). Although the source of EGF in the brain is intrinsic, our discovery of a specific β₂-adrenergic mediation of EGF's central anorexigenic effect suggests a conserved functional interaction at the receptor signaling level across tissues. This potential cross-talk is powerfully supported by the broader paradigm of ErbB-GPCR signaling integration and by direct evidence from cardiac research. A seminal study by Grisanti et al. (2014) demonstrated that transactivation of the EGFR mediated by β-adrenergic receptors is a critical pro-survival pathway in cardiomyocytes, leading to differential activation of ERK1/2 and Akt kinases and decreased apoptosis. This mirrors our hypothesis of receptor-level interaction, showing that β-adrenergic signaling can directly engage and modulate downstream EGFR effector pathways. Thus, by integrating our findings with the established peripheral adrenergic-EGF axis described by Campreciós et al. (2009) and contextualizing them within both the avian monoaminergic framework and the robust evidence of β-AR/EGFR transactivation in other tissues (Grisanti et al., 2014), we propose a convergent mechanism. We posit that EGF fine-tunes appetite regulation by engaging in specific cross-talk with β₂-adrenoceptor signaling, potentially involving transactivation-like events or synergistic modulation of shared intracellular pathways like ERK and Akt, ultimately leading to altered neuronal activity in feeding centers.

The emerging picture from our study and others in avian species points to a highly organized network where anorexigenic signals from growth factors, and other neurotransmitters converge onto specific monoaminergic receptor subtypes—particularly D₁ and β₂—to fine-tune energy balance. This suggests the existence of a sophisticated bidirectional regulatory loop between growth factor and monoaminergic signal transduction mechanisms governing central appetite control.

## Conclusion

In conclusion, central EGF is established here as a potent anorexigenic factor in broiler chicks, acting through highly specific interactions with dopaminergic D₁ and adrenergic β₂ receptors. This reveals previously unrecognized complexity in avian appetite regulation, emphasizing sophisticated cross-talk between growth factor and monoaminergic pathways. Future research should focus on identifying endogenous ligands for the chicken EGF receptor (potentially TGF-α), and mapping the neuroanatomical sites of EGF-monoamine interactions. Additionally, studying how nutritional status or stress modulates this pathway will clarify its physiological significance for avian energy homeostasis.

## Funding

This research did not receive any specific grant from funding agencies in the public, commercial, or not-for-profit sectors.

## Declaration of generative AI and AI-assisted technologies in the writing process

During the preparation of this work the author(s) used Perplexity AI in order to identify and correct potential grammatical errors and improve the overall flow and readability of the manuscript. After using this tool, the author(s) reviewed and edited the content as needed and take(s) full responsibility for the content of the published article.

## CRediT authorship contribution statement

**Zahra Jafari-Ardakan:** Writing – original draft, Data curation. **Morteza Zendehdel:** Writing – original draft, Supervision, Formal analysis, Conceptualization. **Kimia Mahdavi:** Writing – original draft, Methodology.

## Disclosures

The authors report no conflicts of interest.
